# Maternal and congenital syphilis in Indigenous Peoples: a scoping review of the worldwide literature

**DOI:** 10.1186/s12939-023-01890-x

**Published:** 2023-05-09

**Authors:** Andrey Moreira Cardoso, Aline Diniz Rodrigues Caldas, Evelin Santos Oliveira, Enny Santos Paixão, Maria Auxiliadora Santos Soares, Idália Oliveira dos Santos, Maurício Lima Barreto, Maria Yury Travassos Ichihara

**Affiliations:** 1 National School of Public Health, Oswaldo Cruz Foundation, Rio de Janeiro, Estado do Rio de Janeiro, Brazil; 2 Center for Data Integration and Knowledge for Health - CIDACS, Gonçalo Muniz Institute, Oswaldo Cruz Foundation, Salvador, Bahia Brazil; 3 Department of Infectious Disease Epidemiology, London School of Hygiene and Tropical Medicine, London, UK

**Keywords:** Syphilis, Syphilis, congenital, Prenatal care, Infectious Disease Transmission, Vertical, Indigenous Peoples, Health services, indigenous, Indigenous population, Health of indigenous people, Indians, North American, Amerinds, Amerinds, Central American, Indians, South American

## Abstract

**Background:**

Syphilis is among the most common sexually transmitted infections worldwide. When it occurs during pregnancy, it can seriously affect the fetus and newborn`s health. The scarcity of studies on maternal and congenital syphilis in Indigenous Peoples remains an obstacle to its control in these populations. This study aimed to explore the breadth of the literature, map updated evidence, and identify knowledge gaps on maternal and congenital syphilis in Indigenous Peoples worldwide.

**Methods:**

We conducted a Scoping review following Preferred Reporting Items for Systematic Reviews and Meta-Analyses - Extension for Scoping Reviews. In March 2021, we collected data through a priority search on PubMed, Web of Science, Embase, and SciELO.

**Results:**

The strategy yielded 24 studies for analysis. Data in the articles were collected from 1989 to 2020, half from 2015 onwards. Studies were in Oceania and the Americas, mainly in South America (66.7%), particularly in Brazil (50.0%). The topics assessed were Data quality related to maternal and congenital syphilis (20.8%); Diagnosis, provision, access, and use of health services (62.5%); Disease frequency and health inequities (54.2%); Determinants of maternal syphilis and congenital syphilis (20.8%); and Outcomes of maternal and congenital syphilis in the fetus (20.8%). The results show that the available literature on maternal and congenital syphilis is sparse and concentrated in some geographic areas; the frequency of these diseases in Indigenous Peoples varies but is generally higher than in the non-indigenous counterparts; the quality of surveillance data and health information systems is poor; multiple healthcare barriers exist; and the diversity of terms to identify Indigenous Peoples is a challenge to mapping scientific outputs on Indigenous Peoples’ health.

**Conclusions:**

Maternal and congenital syphilis in Indigenous Peoples is a double-neglected condition and research in this area should be given the priority and encouragement it deserves globally. Reliable data and improving access to health care are needed to reduce the burden of syphilis and correctly inform policies and health services response to mitigate ethnic-racial inequalities in maternal and congenital syphilis.

**Supplementary Information:**

The online version contains supplementary material available at 10.1186/s12939-023-01890-x.

## Background

Syphilis is among the most common sexually transmitted infections (STIs) worldwide and is also one of the leading preventable and treatable causes of infant morbidity and mortality [1]. Congenital syphilis, which is transmitted from mother to child during pregnancy, can lead to several adverse fetal outcomes, such as stillbirths, neonatal deaths, preterm births, low birth weight, and congenital infection in infants [1]. The World Health Organization (WHO) has been achieving encouraging results in response to the global initiative to eliminate vertical transmission of syphilis. However, the burden of maternal and congenital syphilis remains substantial and heterogeneous between WHO Regions, countries, and specific population groups [2, 3].

Updated estimates of the burden of maternal and congenital syphilis showed a stable global prevalence of maternal syphilis and a slight decrease in the rate of congenital syphilis (although still high) between 2012 and 2016. These occurred in parallel with an increase in the coverage of antenatal care, screening for syphilis during pregnancy, and treatment of maternal syphilis, although regional disparities persisted [2]. In 2016, for example, while the prevalence of maternal syphilis was 0.10% in Europe, it was 0.86% in the Americas and 1.52% in Africa. Rates of congenital syphilis reached 19, 339, and 1,119 per 100,000 live births, in Europe, the Americas, and Africa respectively.

The disparities in STI and HIV/Aids burden between indigenous and non-indigenous populations are a rising public health concern [3]. Globally, Indigenous Peoples experience low health standards, marginalization, adverse environmental, sanitary, and socioeconomic conditions, and limited healthcare access [4]. The high burden of infectious diseases, high infant and maternal mortality, and low birth weight are among the leading health problems in those populations [4, 5]. This suggests that maternal and congenital syphilis might contribute to the adverse maternal and child health profiles of indigenous populations in several world regions.

A preliminary search carried out by our group of the most relevant scientific databases, including JBI Evidence Synthesis, Cochrane Database of Systematic Reviews, PubMed, Web of Science, Embase, and SciELO, identified two reviews addressing STIs and HIV/Aids in indigenous populations [3, 6]. However, neither review was specifically on maternal and congenital syphilis, one was restricted to publications in English until 2012, and the most recent was limited to Latin America until 2016 [6]. Minichiello and colleagues [3] sought to examine the epidemiology of STIs in indigenous communities worldwide by searching for published and unpublished studies, reports, and guidelines written in English from 2002 to 2012. The authors found 81 studies focused on indigenous populations from high-income countries, neglecting most Indigenous Peoples living in low and middle-income countries. There has been no study reporting syphilis in Indigenous Peoples in North America and Africa. In the other regions, studies that addressed syphilis were sparse and limited to reporting syphilis notification rates or prevalence in the general indigenous population, based on data from surveillance registry or cross-sectional studies respectively. The findings have shown high rates of disease in Indigenous Peoples compared to their counterparts.

More recently, Russell and colleagues [6] carried out a systematic review of the burden of HIV, STI, and viral hepatitis in indigenous and Afro-descendant peoples in 17 countries in Latin America, by searching for published studies and grey literature in English, Spanish and Portuguese, from January 2000 to April 2016. The authors found 62 studies in 12 countries, but only six reported data on maternal and congenital syphilis; four described the prevalence of maternal syphilis through cross-sectional surveys. Data on congenital syphilis were even more limited and restricted to two publications: a Brazilian government report of 17 cases of congenital syphilis among Indigenous Peoples, notified between 2000 and 2005, and a feasibility study for rapid testing of syphilis in remote areas of the Brazilian Amazon, which detected two congenital syphilis and 19 maternal syphilis cases, suggesting limited access to antenatal care, and underreporting of cases. Both the assessed reviews reinforce the global invisibility of maternal and congenital syphilis in Indigenous Peoples. The scarcity of studies on this topic remains an obstacle to estimating ethnic-racial inequalities in the disease burden, identifying its determinants, and defining priority groups for social and health public policy implementation [6].

This scoping review aimed to explore the breadth of the literature, map updated evidence, and identify knowledge gaps on maternal and congenital syphilis in Indigenous Peoples worldwide, to inform future research and social and public health policies.

## Materials and methods

We carried out a scoping review following the Preferred Reporting Items for Systematic Reviews and Meta-Analyses - Extension for Scoping Reviews (PRISMA-ScR) [7] and the Joanna Briggs Institute´s Scoping Reviews guideline [8]. We registered the study protocol on the Open Science Framework (https://osf.io/), at: https://osf.io/2hw8v/?view_only=5ca0f9567d1642758d4c93302a1a2ca9.

We collected data on March 2021 through a search on PubMed (Medline), Web of Science, Embase, and SciELO (Scientific Library Online) bibliographic bases, complemented by a search of the following bases: BVS (Virtual Health Library) and BVS Indígena (Virtual Health Library - Indigenous), LILACS (Latin American and Caribbean Center on Health Sciences Information), OASISBR (Brazilian portal of scientific publications in open access) and BDTD (Digital Library of Theses and Dissertations), as well as manual searching of the bibliographic references of the full-text studies assessed in the eligibility stage. The inclusion criteria were studies addressing syphilis and Indigenous Peoples, with no limits for publication language, dates of data collection or publication, or methods. The exclusion criteria were studies that do not address maternal or congenital syphilis or do not present results separately for Indigenous Peoples and reviews that do not bring additional evidence (such as metanalysis) to that previously reported by the original studies.

### Search strategy

The search terms were identified and improved with the support of a library scientist by finding Medical Subject Headings (MeSH) terms in the Health Sciences Descriptors (DeCS) database (https://decs.bvsalud.org/en/), and by identifying keywords in classic and comprehensive articles related to the topic [3–6, 9, 10]. We combined search terms to meet the inclusion criteria and maximize the sensitivity of the search strategies. We searched terms in titles, abstracts, and keywords in English, Portuguese, and Spanish, whenever applicable. Search terms were minimally adapted to include explicit recommendations on some bibliographic bases (Additional File 1).

### Source of evidence selection

We managed the bibliographic references in the Zotero Standalone software. The selection of studies was performed independently by two reviewers (ESO and ADRC), and a third reviewer (AMC) solved any disagreements. The evidence selection comprised the following steps: (a) Identification – search on bibliographic bases, duplicate exclusion, and manual searching of bibliographic references of the full-text studies assessed in the eligibility step; (b) Screening – studies were checked against the inclusion criteria, by reading the title and abstract; (c) Eligibility – studies were checked against the exclusion criteria, by the full-text reading of the remaining studies in step 2; (d) Inclusion – reporting results of remaining studies in step 3. The sources of evidence excluded following the full-text review are detailed in Additional File 2.

### Data extraction

For data extraction, we used a standardized data extraction form with the following general topics: study identification, objective, study design and population, number of participants investigated, setting, ethnic classification, maternal and congenital syphilis topics covered, the statistical model used for analysis, ethical aspects, measures of frequency and association, determinants of syphilis and outcomes of congenital syphilis, and comments.

During the screening phase, we mapped seven potential domains of interest for analysis related to maternal and congenital syphilis in Indigenous Peoples, which were included in the extraction form: (a) Historical report of gestational and congenital syphilis in indigenous populations; (b) Impacts of social and health public policies on congenital and gestational syphilis in Indigenous Peoples, (c) Diagnosis, provision, access, and use of health services related to gestational and congenital syphilis in Indigenous Peoples, (d) Prevalence or incidence of maternal and congenital syphilis in indigenous populations and health inequalities, (e) Data quality related to congenital and gestational syphilis in Indigenous Peoples, (f) Determinants of congenital or gestational syphilis in indigenous populations, and (g) Outcomes of maternal or congenital syphilis in the conceptus in indigenous populations. Each of these domains could be addressed simultaneously in the same study.

The complete form is presented in Additional File 3.

## Results

### Selection of sources of evidence

**Fig. 1:**
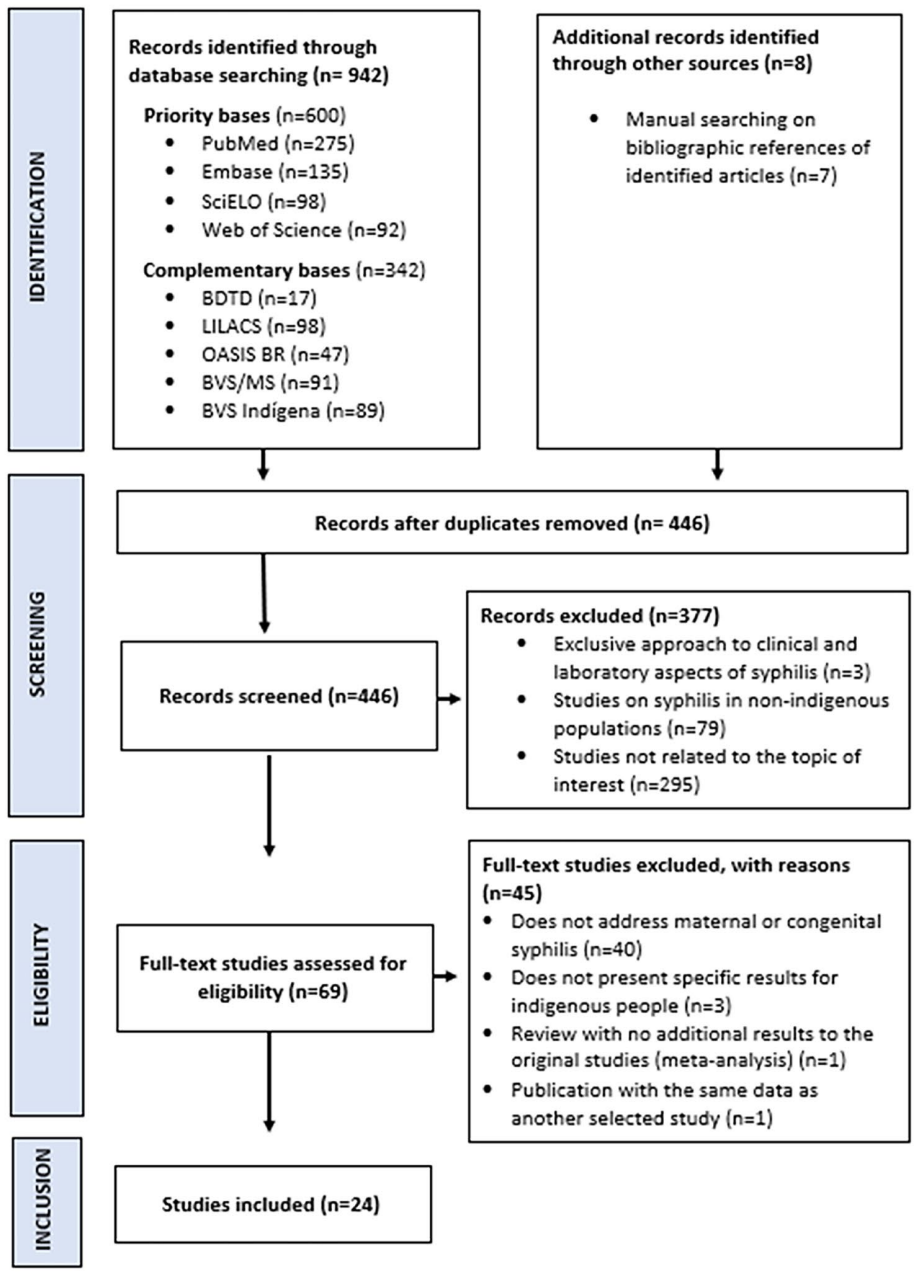
Flow diagram for selection of sources of evidence for the scoping review on maternal and congenital syphilis in Indigenous Peoples

The search strategy yielded 446 unique publications. After title and abstract screening, 69 full articles were read and assessed on eligibility criteria. We finally included 24 studies, two of which were found after a manual search of the bibliographies of the studies identified in the full-text reading (Fig. 1).

### Characteristics and results of individual sources of evidence

**Fig. 2:**
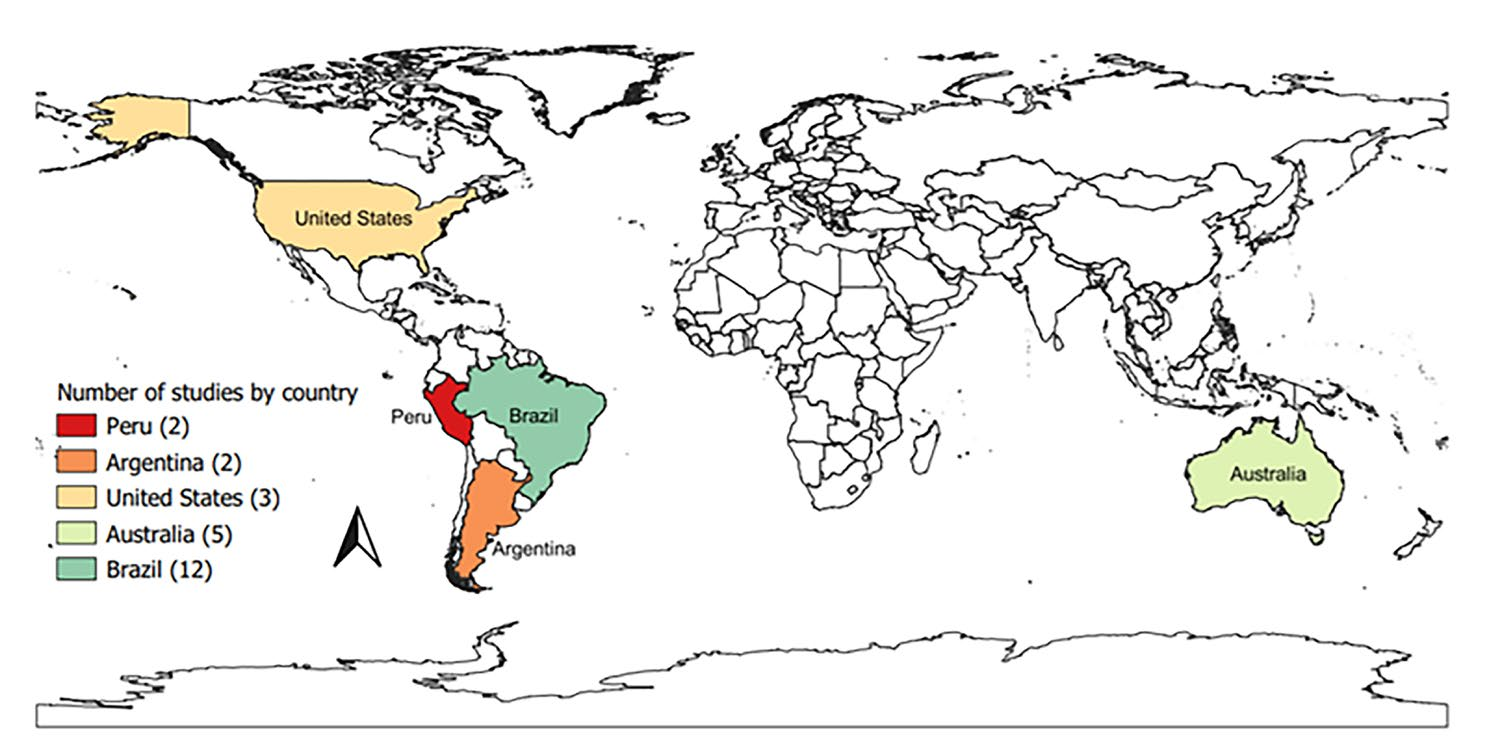
**The number of studies on maternal and congenital syphilis by country in which studies were conducted.** Notes: We downloaded the free shapefile with the countries of the world from the website https://tapiquen-sig.jimdofree.com/english-version/free-downloads/world/. We elaborated the thematic map in the QGIS program (https://www.qgis.org/en/site/)

Data of the selected studies were collected between 1989 and 2020, but in 23/24 collection was from 2000 onwards. Half (12) of the studies collected data from the last five years (Table 1). We only found studies in the Americas and Oceania and most of these were carried out in South America (16/24 or 66.7%), particularly in Brazil (Fig. 2). Most studies consist of papers published in scientific journals (16/24 or 66.7%), in English (17/24, or 70.8%), followed by Portuguese (6, or 25.0%) (Table 1). The most common study design was cross-sectional (seroprevalence) (10–41.7%), followed by qualitative-quantitative designs (case studies + descriptive analysis) (4–16.7%), descriptive ecological design using surveillance data (3–12.5%), and descriptive studies using outbreak data (3–12.5%). One publication reported two study designs to investigate different aspects of syphilis. Eleven (45.8%) studies investigated more than one ethnic group inhabiting the study territory and did not show specific results by specific ethnicity. Three (12.5%) studies focused on indigenous health workers. Of those with indigenous people as a target, 12 (42.8%) defined indigenous ethnicity as residing in an indigenous village or being a member of the community covered by the indigenous health service under investigation, and six (28.6%) used self-declaration of ethnic belonging. A further three did not mention any criteria (Table 1).

**Table 1:** **Characteristics and results of individual sources of evidence.** Scoping review on maternal and congenital syphilis in Indigenous Peoples, 2021.

[Ref. number] First Author (publication year)	Data collection year^1^	Continent^2^; country; region	Public. language^3^	Public. type^4^	Std dsgn^5^	Population; Ethnic determination^6^; participants^7^	Topics covered^8^				
							DQ	DAH	DF	DT	OCS
[11] Cândido (2012)	2007–2010	SA; Brazil; Mato Grosso do Sul state	Pt	MD	E	Several; SD; 344 cases in pw	X		X		
[12] Tiago (2017)	2011–2014	SA; Brazil; Mato Grosso do Sul state	Pt	SP	E	Several; VR; 316 ms, 69 cs	X		X		
[13] Nonato (2020)	2013–2017	SA; Brazil; Rio Branco, Acre state	Pt	SP	DCS	Several; SD; 15 pw	X				
[14] Palma-Pinedo (2012)	2014	SA; Peru; Amazonia	Spn	SP	QLQ	Health workers; NA; 122 health unites and respective health workers	X				
[15] McLeod (2015)	2009–2014	OC; Australia; Northern Territory	En	SP	VR	Several; NI; 31pw	X				
[16] Benzaken (2011)	2009–2011	SA; Brazil; Amazonia	En	CA	CS	Several; VR; 3650 pw		X			
[17] Carvalho (2011)	2009–2011	SA; Brazil; Amazonia	En	CA	CE	Several; VR; NI		X			
[18] Domingues (2014)	2011–2012	SA; Brazil; National	En	SP	CS	Several; SD; 99 pw		X	X		
[19] Garnelo (2019)	2008–2009	SA; Brazil; National	Pt	SP	CS	Several; SD; 3.967 pw		X			
[20] Picoli (2020)	2018	SA; Brazil; Mato Grosso do Sul state	Pt	SP	QLQ	Health workers; NA; 33 nurses from the indigenous health system		X			
[21] Schmeing (2012)	2012	SA; Brazil; Amambai, Mato Grosso do Sul state	Pt	MD	QLQ	Health workers; NA; 24 health workers		X			
[22] Ruffinen (2015)	2008	SA; Brazil; Amazônia, Alto Solimões	En	SP	QLQ	Ticuna; MPC; 6473 indigenous (pw not informed)		X	X		
[23] Da Costa Ribeiro (2014)	2009–2011	SA; Brazil; Amazônia	En	CA	CS	Several; NI; 967w (81,1% indigenous)		X	X		
[24] CDC (2010)	2007–2009	NA; EUA; Arizona	En	RP	D	Southwest Indian Nation; MPC; 106 cases (6 cs)		X			X
[25] Bowen (2018)	2013–2015	NA; EUA; NI	En	SP	D	American Indian/Alaska Native; VR; 136 cases (2 cs)		X	X		
[26] Miele (2020)	2014–2018	NA; EUA; National	En	CA	E	American Indian/ Alaska Native; NI; 35 cs		X	X		X
[27] Bornay-Llinares (2014)	2012–2013	SA; Argentina; Missiones	En	CA	CS	Guarani-Mbya; VR; 652 (342w, 250 < 10yo)		X	X		
[28] Marx (2020)	NI	SA; Argentina; Puerto Iguazú	En	SP	CS	Mbyá-Guarani; VR; 551 (90 children)		X		X	
[29] Bright (2016)	2011–2015	OC; Australia; Queensland, North. Territ. & Western Australia	En	SP	D	Aboriginal and Torres Strait Islanders; VR; 790 cases (7 cs)		X	X		
[30] How (1994)	1989–1991	OC; Australia; North West Mildura	En	SP	CS	Aboriginal; MPC; 71pw		X	X	X	X
[31] Benzaken (2017)	2009–2011	SA; Brazil; Amazonia	En	SP	CS	Several; VR; 3650 pw			X	X	
[32] Panaretto (2006)	2000–2003	OC; Australia; Townsville	En	SP	CS; CH	Aboriginal and Torres Strait Islander; SD; 432 pw			X	X	X
[33] Ormaeche (2012)	2007–2008	SA; Peru; Amazonia	En	SP	CS	Several; VR; 1251 pw, 697 partners			X	X	
[34] Burton (2019)	2011–2014	OC; Australia; Northern Territory	En	SP	CC	Northern Territory Indigenous births; SD or BR; 380 cases (preterm birth) and 380 controls					X

Notes: Data collection year: NI = Not informed. 2- Continent: SA = South America; NA = North America; OC = Oceania. 3- Publication language: En = English; Pt = Portuguese; Spn = Spanish. 4- Publication type: MD = Master dissertation; PhD = PhD thesis; SP = Scientific paper; CA = Conference/Congress abstract; RP = Report; REV = Review (Systematic, others, meta-analysis). 5- Study design: DCS = Descriptive, case series; CS = Cross-Sectional/Seroprevalence; E = Ecological, descriptive using surveillance data; D = Descriptive, outbreaks; CC = Case-Control; CH = Cohort; SR = Systematic review or meta-analysis; CT = Community trial; VR = Validity/reliability; CE = Costs-effectiveness (screening); QLQ = Qualitative/Quantitative (Case study and cross-sectional). 6- Ethnic determination: NA = not applicable; VR = residence in the indigenous village; SR = self-report; BR = birth registration data; MPC = member of the population covered by the health service; NI = Not informed. 7- Number of participants: pw = pregnant women, w = women, ms = maternal syphilis; cs = congenital syphilis; NI = Not informed. 8- Topics covered: HT = Historical report of gestational and congenital syphilis in indigenous populations; DQ = Data quality related to congenital and gestational syphilis in Iindigenous Ppeoples; DAH = Diagnosistic, provision, access, and use of health services related to gestational and congenital syphilis in Iindigenous Ppeoples; DF = Prevalence or Incidence of maternal and/or congenital syphilis in indigenous populations, and health inequities; DT = Determinants of congenital or gestational syphilis in indigenous populations; OCS = Outcomes of maternal or congenital syphilis in the conceptus in indigenous populations; and IP = Impacts of social and health public policies on congenital and gestational syphilis in indigenous populations.

No study addressed (a) Historical report of gestational and congenital syphilis in indigenous populations or (b) Impacts of social and health public policies on congenital and gestational syphilis in Indigenous Peoples, while the other five domains were addressed in one or more studies: nine (37.5%) addressed only one domain, while 12 (50.0%), two (8.3%) and one (4.2%) addressed simultaneously two, three or four domains, respectively (Table 1). The most investigated domains were (c) Diagnosis, provision, access, and use of health services (15/24, 62.5%) and (d) Prevalence or Incidence and health inequalities (13/24, 54.2%). All five (20.8%) studies on the syphilis determinants domain investigated maternal syphilis through a cross-sectional design (seroprevalence) to estimate prevalence odds ratios through multiple logistic regression. We did not identify any studies addressing determinants of congenital syphilis (Table 2).

**Table 2:** Study design, outcome, statistical analysis, measures of association, and exposures independently associated with indigenous maternal syphilis or syphilis in indigenous adults, 1989–2020.

Study identification	Study design	Outcome	Statistical model for analysis	Measures of association	Factors statistically associated (adjusted or crude^#^)
[28] Marx, 2020	Cross-Sectional	Syphilis in indigenous adults (men and women)	Logistic Regression	Prevalence OR	- None
[30] How, 1994	Cross-Sectional	Indigenous Maternal syphilis	Logistic Regression	Prevalence OR	- Marital status (single) ^**#**^
[31] Benzaken, 2017	Cross-Sectional	Syphilis in indigenous adults (men and women)	Multiple Logistic Regression	Prevalence OR	- Sex (men) - Age (older) - High level of intrusion^¶^ - Moderate and high levels of population mobility
[32] Panaretto, 2006	Cross-Sectional	Indigenous maternal STI*	Multiple Logistic Regression	Prevalence OR	- Age < 20 years - Hazardous/harmful use of alcohol - Unwanted pregnancy
[33] Ormaeche, 2012	Cross-Sectional	Indigenous Maternal syphilis	Multiple Logistic Regression	Prevalence OR	- Partner with syphilis

*STI (C. trachomatis, N. gonorrhea, T. vaginalis, and syphilis) in pregnant indigenous women

# Crude association

¶Defined as presence of the timber industry, agro-business, mining activities, or areas of “garimpo”, or informal mining, inside or near the área

## Synthesis of results

### Data quality related to congenital and gestational syphilis in Indigenous Peoples (DQ)

Five studies (20.8%) assessed the quality of information systems data related to maternal and congenital syphilis in Indigenous Peoples; three of these were in Brazil [11–13], one in Peru [14], and one in Australia [15]. The quality dimensions addressed were completeness [11, 13], coverage [11, 12, 15], and consistency [15], while one study addressed factors associated with poor quality of syphilis information systems [14].

Completeness of the variable race/skin color, whereby indigenous people are identified in Health Information Systems in Brazil, and other variables of the maternal syphilis notification form, such as education, occupation, clinical classification, and treatment of the partner, was heterogeneous [11]. In the National Network of Public Health Laboratories database in Brazil, race/skin color was only completed in 50.4% of the tests for maternal syphilis and 60.3% of the tests for congenital syphilis [13].

Coverage was analyzed through the linkage of different laboratory and case notification databases, showing 12.7% underreporting of syphilis cases in pregnant women in the general population of the Brazilian state of Mato Grosso do Sul [11]. Also, another study conducted a specific linkage between the Indigenous Health Care Information System and the Brazil Notifiable Diseases Information System (SINAN) for Syphilis, in this state, revealing an underreporting of 57.0% of maternal syphilis and 47.0% of congenital syphilis in SINAN – Syphilis [12].

The consistency of the maternal and congenital syphilis notification was analyzed in Australian Indigenous Peoples, showing an inappropriate diagnosis of 44.0% of pregnant women and 95% of newborns with syphilis, resulting in a sixfold increase in the congenital syphilis incidence rate estimated through uncorrected data [15].

A study in the Peruvian Amazon showed that poor data quality on congenital syphilis in Indigenous Peoples was associated with barriers related to the following domains: human resources - lack of human resources, insufficient training and high staff turnover, lack of common mechanisms to ensure data reliability; failures of the health system itself - low budget, fragmentation or competition between different health information systems, lack of resources for screening and diagnosis; and socio-cultural barriers [14].

### Diagnosis, provision, access, and use of health services related to gestational and congenital syphilis in Indigenous Peoples (DAH)

Fifteen studies (62.5%) assessed the diagnosis, provision, access, and use of health services related to maternal and congenital syphilis in Indigenous Peoples, eight of them in Brazil [16–23], three in the United States [24–26], two in Argentina [27, 28], and two in Australia [20, 29].

Prenatal care coverage is generally low in indigenous populations [18, 21], and access to laboratory resources is quite limited [16, 17, 20, 22], with missed opportunities for diagnosis and treatment [20, 22, 26]. All studies showed an under-detection and under-reporting of cases. The studies indicated that the offer of massive rapid testing [16, 23], the increase in general and gestational screening for syphilis [16], health staff training [20, 30], intersectoral articulation, and coordinated response with community participation [24, 25, 29] are key points for the control of syphilis in these populations, particularly in remote areas [17].

### Incidence and prevalence of congenital and gestational syphilis in indigenous populations and health inequities (DF)

Thirteen studies (54.2%) assessed the frequency of maternal and congenital syphilis in Indigenous Peoples, six of them in Brazil [11, 12, 18, 22, 23, 31], three in Australia [29, 30, 32], two in the United States [25, 26], and one in Peru [33], and Argentina [27]. Most studies (8/13; 61.5%) reported results of seroprevalence surveys in pregnant women [18, 22, 23, 31–33] or children [27], and 3/13 (23.1%) reported detection rates of syphilis in pregnant women [12] or incidence rates of congenital syphilis in live births [12, 29], based on surveillance data. Three descriptive studies (23.1%) with surveillance or outbreak data reported only absolute case frequencies [11, 25, 26].

The average prevalence of maternal syphilis in the Brazilian Amazon was 1.52% (95%CI: 1.14 − 1.89), with heterogeneity among nine of the 34 Special Indigenous Health Districts (DSEI) that make up the Indigenous Health Care Subsystem in Brazil, in which the prevalence ranged from zero (DSEI Medium Purus and DSEI Parintins) to 3.38% (95%CI: 0.46–6.30) (DSEI Vale do Javari) [31]. The prevalence reported in other studies in the Brazilian [22, 23], and Peruvian Amazon [33], as well as in Australia [32], varied within the same range. Extremely low (0.55%)[18] and extremely high (28.0%)[30] prevalences were reported in two studies with convenience samples.

Only three studies reported congenital syphilis [12, 27, 29]. Bornay-Llinares and colleagues [27] reported a prevalence of 1.6% in children from 19 Guarani villages in Argentina. The incidence rate of congenital syphilis in Mato Grosso do Sul state, Brazil, was 1,070/100,000 live births [12], which is substantially higher than that reported in an outbreak in Australia, corresponding to 23/100,000 [29], and similar to that reported in Africa (1,119 per 100,000), in 2016 [2].

The studies show heterogeneity in the frequency of these outcomes in the different contexts investigated. The remote location implies increased risk due to limited healthcare access, even though the proximity of urban areas confers an additional risk of infection [33].

### Determinants of congenital or gestational syphilis in indigenous populations (DT)

Five studies (20.8%) assessed determinants of syphilis in indigenous populations, two in Australia [30, 32], and one in each of the following countries: Brazil [31], Peru [33], and Argentina [28]. Three studies investigated factors specific to maternal syphilis [30, 32, 33], and two identified factors associated with syphilis in general that directly affect the likelihood of gestational and congenital syphilis [28, 31]. No study investigated factors associated with congenital syphilis (Table 2).

All the studies were cross-sectional and used logistic regression to estimate prevalence odds ratios. Benzaken and colleagues [31] carried out a seroprevalence survey on 45,967 indigenous people from 83 remote villages in the Brazilian Amazon. Contextual factors remained significantly associated with syphilis in the adjusted model; these included high levels of intrusion into indigenous territories (high: OR: 1.80, 95%CI: 1.49–2.18) and elevated population mobility (moderate: OR 7.46, 95%CI 2.69–20.67; high: OR 15.95, 95%CI: 5.96–42.71). Single women were more likely to have syphilis in pregnancy [30], as well as those in younger age groups [28, 32], those whose sexual partners had syphilis [33], and pregnant women who reported alcohol use and unwanted pregnancy [32].

### Outcomes of maternal or congenital syphilis in the conceptus in indigenous populations (OCS)

Five studies (20.8%) assessed outcomes of maternal or congenital syphilis in the conceptus in indigenous populations: three in Australia [30, 32, 34], and two in the United States [24, 26]. Three studies used analytical designs, two were prospective cohorts in pregnant indigenous women [31, 32], and one was a retrospective, hospital-based case-control study [34]. The other two studies were descriptive, with outbreak investigation data [24] and routine surveillance data [26].

Syphilis during pregnancy was associated with preterm birth in indigenous pregnant women in Australia (Odds Ratio: 21.5; 95%CI 2.26–205.0) [30]. In the United States, 37% of 35 indigenous newborns with congenital syphilis reported to the Centers for Disease Control and Prevention (CDC) in 2018 were preterm [26]. In the case-control study carried out by Burton and colleagues [34] in Australia, the absence of syphilis among the few preterm births precluded estimated measures of association.

In Australia, indigenous pregnant women with syphilis had a relative risk of 4.0 (95% CI 2.56–6.25) for stillbirth [30]. In the United States, a study reported two stillbirths among six congenital syphilis cases identified during a syphilis outbreak in indigenous populations. A second study identified four stillbirths among 35 congenital syphilis cases notified to CDC in 2018 [26].

In a cohort of indigenous pregnant women in Australia, the perinatal mortality rate in women with gestational syphilis (182/1,000 live births) was about 7.5 times the corresponding rate in pregnant women without STI (24/1000 live births) [32]. In the United States, Miele and colleagues [26] reported one early infant death among 35 congenital syphilis cases in indigenous people notified to the CDC in 2018. Panaretto and colleagues [32] reported a prevalence of low birth weight in children of pregnant women with syphilis (36.4%) about three times the prevalence recorded in children of pregnant women without STI (11.1%) in Australia.

## Discussion

### Summary of evidence

Our scoping review process identified 24 publications that have addressed maternal and congenital syphilis in Indigenous Peoples worldwide. We observed a substantial increase in publications on this topic, 50% of which were published in the last five years. This is in line with the persistence of a high burden of syphilis-related disease globally [2], and with WHO goals for eliminating congenital syphilis [1], although we did not find a specific mention of this issue in Indigenous Peoples in these publications.

The high proportion of studies conducted in Brazil (50%) together with the absence of studies in many countries that have a significant indigenous population and effective indigenous participation in decision-making processes in health and definition of research priorities are noteworthy. The high frequency of studies in Brazil seems to result from the promotion and encouragement of regional research through the implementation of a science and technology unit of the Ministry of Health – the Oswaldo Cruz Foundation (FIOCRUZ), in the state of Mato Grosso do Sul [12, 20]. This institution offered a professional master’s course in border health surveillance in the region [11, 21]. Another contributing factor was the conducting of extensive research to analyze the cost-effectiveness of implementing rapid tests for syphilis and HIV in indigenous populations in remote areas, where access to health and laboratory networks is minimal [16, 17, 22, 23, 31].

The absence of studies in countries with a substantial indigenous population, such as China, India, Indonesia, Ethiopia, Philippines, Canada, Mexico, and New Zealand, might be due to low scientific interest in the subject, use of non-indexed descriptors, or nonspecific keywords in publications, and grey literature not identified in searches. Other possible explanations include concern about further stigmatizing this population, lack of prioritization of this as an issue, and limited capacity to self-conduct and report on the data.

According to the United Nations, even though the most accepted criterion for defining indigenousness is self-identification, there is no consensus on a single defining characteristic or term, given the enormous diversity of traditional peoples and societies. Although the use of the word “indigenous” is widespread, it has a negative connotation in some regions. In this case, it is usually exchanged for other words, such as tribes, first peoples/nations, aboriginals, and ethnic groups [35], which makes it difficult to identify all relevant studies on a purely bibliographic basis. However, our review was exhaustive in including these terms in search strategies.

The studies which investigated data quality related to maternal and congenital syphilis in Indigenous Peoples (DQ) analyzed three quality dimensions: completeness, coverage, and consistency. The quality of the records was found to be poor, showing underreporting of cases and incompleteness of notifications, particularly for socioeconomic, treatment, and ethnicity variables, which can lead to information or selection biases. The poor data quality contributes to underestimating the burden of these diseases in Indigenous Peoples and reduces accuracy in estimating ethnic-racial inequities and identifying social determinants of syphilis; this in turn limits the usefulness of the data to inform social and health policies for disease control [11, 15]. As in the report by Palma-Pinedo and colleagues [14] specifically for syphilis in the Peruvian Amazon, Sousa and colleagues [36] highlighted the poor quality of indigenous data in the health information systems in Brazil. This was reported to be due to the high turnover of the workforce, lack of training, work overload, and lack of integration between different information systems. Such evidence indicates the need for investment in the expansion of financial, material, and workforce resources, training for syphilis diagnosis, management and surveillance, awareness of health workers of intercultural work, and strengthening of surveillance systems to improve the quality of health care and information systems, including those specifically for syphilis.

The studies addressing the diagnosis, offer, access, and use of health services by Indigenous Peoples (DAH) highlighted existing barriers to health care, such as low prenatal care coverage and limited laboratory resources for diagnosis. These conditions are responsible for missed opportunities for diagnosis and treatment, resulting in under-detection and under-reporting of maternal and congenital syphilis. The intersection of several geopolitical and health care management levels and heterogeneous levels of health care organization, particularly in remote areas, are strong limitations for surveillance and timely diagnosis and treatment, creating further difficulties for the control and prevention of congenital syphilis. Syphilis control in these populations, particularly in remote areas [17, 37], presupposes the expansion of access to health care through universal systems, with an emphasis on primary care, especially prenatal care and delivery, which is socio-culturally sensitive [19]. It includes the offer of rapid testing for syphilis and other STIs [16, 23], the expansion of screening for syphilis in the general population, in the most vulnerable groups and during pregnancy and childbirth [16, 27], health worker training [20, 30], intersectoral articulation and coordinated responses, with community participation [24, 25, 29].

As for the frequency of maternal and congenital syphilis in Indigenous Peoples (DF), most studies reported the prevalence of syphilis based on seroprevalence surveys, and only three studies reported maternal syphilis detection rates or incidence of congenital syphilis. There was heterogeneity in the frequency of outcomes between different ethnic groups, territories, regions, and countries, with a higher burden of disease in comparison to their counterparts [12, 31], as widely documented for other diseases and conditions [4]. The location in regions with limited access to health care, on the international border, or in remote areas, proved to be a relevant context for the high frequency of maternal and congenital syphilis [11, 22] a fact also reported by Hierink and colleagues [37] about other infectious diseases in low- and middle-income countries. However, the comparison of frequencies between indigenous populations in remote and urban areas in the same region suggests that proximity to urban areas may be an additional risk for the transmission of syphilis in these populations [33]. The results indicate that the life context of Indigenous Peoples plays a central role in the frequency of maternal and congenital syphilis, influencing the infectivity, transmission rate, and duration of the disease [37, 38].

Studies on syphilis determinants in indigenous populations (DT) are scarce. All identified studies had a cross-sectional design and reported factors associated with maternal syphilis. No study investigated factors associated with congenital syphilis. In general, factors associated with maternal syphilis were similar to those described in the literature for the non-indigenous population [28, 30, 32, 33]. Contextual factors of particular relevance to Amazonian Indigenous Peoples were highlighted, such as the high degree of intrusion in indigenous territories, including illegal mining or deforestation, and the increased mobility of the population to access resources in regional attraction centers [31]. A recent analysis of the risk of the geographic spread of COVID-19 in Indigenous Peoples in Brazil, using proxy indicators of mobility and intrusion in indigenous territories, confirmed these context-related vulnerabilities to infectious diseases [39, 40]. The lack of a significant association with other supposed risk variables is possibly attributed to the low power of the studies due to the relatively low number of events of interest in small populations.

Gestational or congenital syphilis in indigenous people (OCS) was significantly associated with the outcomes of prematurity, stillbirth, perinatal mortality, and low birth weight [24, 26, 30, 32, 34]. It is noteworthy that indigenous populations, in general, constitute small population groups [41], thus the absolute frequency of health outcomes related to syphilis tends to be neglected. The rarity of events of interest in health investigations often results in methodological challenges, given the low power of studies to investigate disease determinants.

### Knowledge gaps and future research

The few existing publications on the frequency of maternal and congenital syphilis are mainly based on prevalence data and seem somewhat biased due to underreporting of the disease, lack of comprehensive data quality, and restriction of analysis to some countries in the Americas and Oceania. Future research should provide robust estimates of the frequency of maternal and congenital syphilis in Indigenous Peoples in a wide range of populations and contexts to better represent diversity, vulnerability, and inequalities. Performing routine analyses of data quality, estimating underreported cases, and generating possible correction factors at different geographic scales could support the monitoring of ethnic-racial trends in syphilis inequalities. The linkage of datasets addressing all phases of the health-disease process could improve data completeness and consistency, enabling innovative and robust analysis of determinants and impacts of public policies for controlling the disease. It is also necessary that health and surveillance systems be improved by continuously increasing the quality of health information systems data, availability of up-to-date and reliable sociodemographic data, and eliminating access barriers to universal, public healthcare provided by qualified professionals.

Theoretical models for analyzing the determinants of maternal and congenital syphilis in Indigenous Peoples must consider contextual variables that enable the identification of modifiable social determinants beyond those already well-established for the general population to support social and health policies implemented at the state level. The investigation of incident outcomes (maternal and congenital syphilis), possibly prioritizing analytical epidemiological designs, could lead to risk estimates and a better understanding of causality and consistently monitor the temporal trend of the disease. Research on syphilis outcomes in the conceptus is also scarce and deserves to be expanded into different contexts. Assessment of the impact of social and health policies on maternal and congenital syphilis and its outcomes in the conceptus is non-existent in the accessed literature and should be considered a hotspot for future research.

## Limitations

Although the United Nations estimates there are more than 476 million indigenous people in the world, spread across 90 countries and making up 6.2% of the world population [42], we were only able to identify studies on this topic in five countries in two continents. It is worth mentioning that although we designed an exhaustive search to identify literature on Indigenous Peoples, we recognize that, given the heterogeneity of names and ways of reporting studies on Indigenous Peoples, there was a potential loss of studies. However, although there may have been some selection and publication bias, this is a limitation that is difficult to overcome unless the recommendations for using standardized terms to register these studies are adopted globally. Further, the similarity between the evidence collected in our review and that of the global literature on indigenous people’s health, and the consistency between the results of the studies included in the review, corroborate the robustness of our findings.

## Conclusions

The results of this study have shown that (a) the available literature on maternal and congenital syphilis is sparse and concentrated in some geographic areas; (b) the frequency of maternal and congenital syphilis in Indigenous Peoples varies but is generally higher than in the non-indigenous counterparts; (d) the quality of surveillance data and health information systems is poor; (e) multiple healthcare barriers exist such as the low quality of prenatal care and childbirth, inappropriate screening strategies for syphilis, insufficient funding for health systems, poor organization and articulation of health services and incipient social participation; (f) the diversity of terms to identify Indigenous Peoples is a challenge to mapping scientific outputs on Indigenous Peoples’ health.

Maternal and congenital syphilis in Indigenous Peoples represents a double-neglected condition and research in this area should be given the priority and encouragement it deserves globally. Indigenous Peoples have historically been exposed to discrimination, violence, and social exclusion, entailing greater vulnerability to several diseases, including syphilis. Thus, reliable data and improved access to health are needed to reduce the burden of syphilis and correctly inform policy and service responses to mitigate ethnic-racial inequalities in maternal and congenital syphilis.

## Electronic supplementary material

Below is the link to the electronic supplementary material.

Additional File 1: Detailed search strategy in all databases.

Additional File 2: Sources excluded following full-text review.

Additional File 3: Data extraction instrument.

## Data Availability

Not applicable.

## Abbreviations

STIs: Sexually Transmitted Infections
WHO: World Health Organization
PRISMA- ScR: Preferred Reporting Items for Systematic Reviews and Meta-Analyses - Extension for Scoping Reviews
SciELO: Scientific Library Online
BVS: Virtual Health Library
BVS: Indígena Virtual Health Library - Indigenous
LILACS: Latin American and Caribbean Center on Health Sciences Information
OASISBR: Brazilian Portal of Scientific Publications in Open Access
BDTD: Digital Library of Theses and Dissertations
MeSH: Medical Subject Headings
DeCS: Health Sciences Descriptors
DSEI: Special Indigenous Health Districts
SINAN: Brazil Notifiable Diseases Information System
DSEI: Special Indigenous Health Districts
CDC: Centers for Disease Control and Prevention
FIOCRUZ: Oswaldo Cruz Foundation
