# Supplementary material for: Maternal and congenital syphilis in Indigenous Peoples: a scoping review of the worldwide literature
Source: Int J Equity Health. 2023 May 9;22:84. doi: 10.1186/s12939-023-01890-x (PMC10169209; doi:10.1186/s12939-023-01890-x)
Supplement: Supplementary file 1 — Additional File 1: Detailed search strategy in all databases. [file 12939_2023_1890_MOESM1_ESM.pdf]

Additional File 1: Detailed search strategy in all databases

Web of Science

Maternal syphilis: (#1 AND #2).

Strategy#1: ((syphilis[Title/Abstract]) OR (syphilis[MeSH Terms]) OR (“treponemal infections”[MeSH Terms]) OR (treponema[Title/Abstract]) OR (LUES[Title/Abstract]) OR (LUES[MeSH Terms]))

Strategy#2: ((pregnancy[Title/Abstract]) OR (“Pregnancy Complications, Infectious”[MeSH Terms]) OR (pregnant*[Title/Abstract]) OR (maternal*[Title/Abstract]) OR (maternity[Title/Abstract]) OR (mothers[MeSH Terms]) OR (mother*[Title/Abstract]) OR (women[Title/Abstract]) OR (woman[Title/Abstract]) OR (parturient*[Title/Abstract]) OR (gestational[Title/Abstract]) OR (prenatal*[Title/Abstract]) OR (antenatal[Title/Abstract]) OR (“vertical transmission”[Title/Abstract])) OR (“Vertical Infection”[Title/Abstract])) OR (congenital[Title/Abstract])) OR (congenital[MeSH Subheading])) OR (“mother-to-child transmission”[Title/Abstract])) OR (“Infectious Disease Transmission, Vertical”[MeSH Terms])))

Congenital syphilis (#3)

Strategy#3: ((“Syphilis, Congenital”[MeSH Terms]) OR (“congenital syphilis”[Title/Abstract]))

Indigenous Peoples (#4)

Strategy#4: ((indian*[Title/Abstract]) OR (“indigenous peoples”[MeSH Terms])) OR (“population groups”[MeSH Terms])) OR (“indigenous culture”[MeSH Terms])) OR (“health services, indigenous”[MeSH Terms])) OR (“oceanic ancestry group”[MeSH Terms])) OR (“alaska natives”[MeSH Terms])) OR (“indigenous population”[Title/Abstract])) OR (“health of indigenous people”[Title/Abstract])) OR (“native americans”[Title/Abstract])) OR (“Indians, North American”[MeSH Terms])) OR (Inuits[MeSH Terms])) OR (“first nations”[Title/Abstract])) OR (amerinds[Title/Abstract])) OR (“Amerinds, Central American”[MeSH Terms])) OR (“Indians, South American”[MeSH Terms])) OR (Tribes[Title/Abstract])) OR (indigen*[Title/Abstract])) OR (aborigine[Title/Abstract])) OR (tribal[Title/Abstract])) OR (“native people”[Title/Abstract])).

Final strategy: Maternal and congenital syphilis in Indigenous Peoples (((#1 AND #2) OR #3) AND #4).

Embase

Maternal syphilis: (#1 AND #2).

Strategy#1: (syphilis:ti,ab,kw OR treponema:ti,ab,kw OR treponematosis:ti,ab,kw)

Strategy#2: (pregnancy:ti,ab,kw OR 'pregnancy complications, infectious':ti,ab,kw OR pregnant*:ti,ab,kw OR maternal*:ti,ab,kw OR maternity:ti,ab,kw OR mothers:ti,ab,kw OR mother*:ti,ab,kw OR women:ti,ab,kw OR woman:ti,ab,kw OR parturient*:ti,ab,kw OR gestational:ti,ab,kw OR prenatal*:ti,ab,kw OR antenatal:ti,ab,kw OR 'vertical transmission':ti,ab,kw OR 'vertical infection':ti,ab,kw OR congenital:ti,ab,kw OR 'mother to child transmission':ti,ab,kw)

Congenital syphilis (#3)

Strategy#3: ('syphilis, congenital':ti,ab,kw OR 'congenital syphilis':ti,ab,kw)

Indigenous Peoples (#4)

Strategy#4: ('indigenous peoples':ti,ab,kw OR 'population groups':ti,ab,kw OR 'indigenous culture':ti,ab,kw OR 'health services, indigenous':ti,ab,kw OR 'oceanic ancestry group':ti,ab,kw OR 'alaska natives':ti,ab,kw OR 'indigenous population':ti,ab,kw OR 'continental population groups':ti,ab,kw OR 'health of indigenous people':ti,ab,kw OR 'native americans':ti,ab,kw OR inuits:ti,ab,kw OR 'first nations':ti,ab,kw OR amerinds:ti,ab,kw OR tribes:ti,ab,kw OR indigen*:ti,ab,kw OR aborigine:ti,ab,kw OR tribal:ti,ab,kw)

Final strategy: Maternal and congenital syphilis in Indigenous Peoples (((#1 AND #2) OR #3) AND #4).

Pubmed

Maternal syphilis: (#1 AND #2).

Strategy#1:((syphilis[Title/Abstract]) OR (syphilis[MeSH Terms]) OR ("treponemal infections"[MeSH Terms]) OR (treponema[Title/Abstract]) OR (LUES[Title/Abstract]) OR (LUES[MeSH Terms]))

Strategy#2: ((pregnancy[MeSH Terms]) OR (pregnancy[Title/Abstract]) OR ("Pregnancy Complications, Infectious"[MeSH Terms]) OR (pregnant*[Title/Abstract]) OR (maternal*[Title/Abstract]) OR (maternity[Title/Abstract]) OR (mothers[MeSH Terms]) OR (mother*[Title/Abstract]) OR (women[Title/Abstract]) OR (woman[Title/Abstract]) OR (parturient*[Title/Abstract]) OR (gestational[Title/Abstract]) OR (prenatal*[Title/Abstract]) OR (antenatal[Title/Abstract]) OR ("vertical transmission"[Title/Abstract]) OR ("Vertical Infection"[Title/Abstract]) OR (congenital[Title/Abstract]) OR (congenital[MeSH Subheading]) OR ("mother-to-child transmission"[Title/Abstract]) OR ("Infectious Disease Transmission, Vertical"[MeSH Terms]))

Congenital syphilis (#3)

Strategy#3: (("Syphilis, Congenital"[MeSH Terms]) OR ("congenital syphilis"[Title/Abstract]))

Indigenous Peoples (#4)

Strategy#4: ((indian*[Title/Abstract]) OR ("indigenous peoples"[MeSH Terms]) OR ("population groups"[MeSH Terms]) OR ("indigenous culture"[MeSH Terms]) OR ("health services, indigenous"[MeSH Terms]) OR ("oceanic ancestry group"[MeSH Terms]) OR ("alaska natives"[MeSH Terms]) OR ("indigenous population"[Title/Abstract]) OR ("health of indigenous people"[Title/Abstract]) OR ("native americans"[Title/Abstract]) OR ("Indians, North American"[MeSH Terms]) OR (Inuits[MeSH Terms]) OR ("first nations"[Title/Abstract]) OR (amerinds[Title/Abstract]) OR ("Amerinds, Central American"[MeSH Terms]) OR ("Indians, South American"[MeSH Terms]) OR (Tribes[Title/Abstract]) OR (indigen*[Title/Abstract]) OR (aborigine[Title/Abstract]) OR (tribal[Title/Abstract]) OR ("native people"[Title/Abstract]))

Final strategy: Maternal and congenital syphilis in Indigenous Peoples (((#1 AND #2) OR #3) AND #4).

Pubmed, Scielo, Lilacs e OASIS BR

Maternal syphilis: (#1 AND #2).

Strategy#1:((syphilis[Title/Abstract]) ("treponemal infections"[Title/Abstract]) OR (LUES[Title/Abstract]))

Strategy#2: ((pregnancy[Title/Abstract]) OR ("Pregnancy Complications, Infectious"[Title/Abstract]) OR (pregnant[Title/Abstract]) OR (maternal[Title/Abstract]) OR (maternity[Title/Abstract]) OR (mother[Title/Abstract]) OR (women[Title/Abstract]) OR (woman[Title/Abstract]) OR (parturient[Title/Abstract]) OR (gestational[Title/Abstract]) OR (prenatal[Title/Abstract]) OR (antenatal[Title/Abstract]) OR ("vertical transmission"[Title/Abstract]) OR ("Vertical Infection"[Title/Abstract]) OR (congenital[Title/Abstract]) OR ("mother-to-child transmission"[Title/Abstract]) OR ("Infectious Disease Transmission" "[Title/Abstract] OR Vertical"[Title/Abstract]))

Congenital syphilis (#3)

Strategy#3: (("Syphilis, Congenital"[Title/Abstract]) OR ("congenital syphilis"[Title/Abstract]))

Indigenous Peoples (#4)

Strategy#4: ((indian[Title/Abstract]) OR ("indigenous peoples"[Title/Abstract]) OR ("population groups"[Title/Abstract]) OR ("indigenous culture"[Title/Abstract]) OR ("health services, indigenous"[Title/Abstract]) OR ("oceanic ancestry group"[Title/Abstract]) OR ("alaska natives"[Title/Abstract]) OR ("indigenous population"[Title/Abstract]) OR ("health of indigenous people"[Title/Abstract]) OR ("native americans"[Title/Abstract]) OR ("Indians, North American"[Title/Abstract]) OR (Inuits[Title/Abstract]) OR ("first nations"[Title/Abstract]) OR (amerinds[Title/Abstract]) OR ("Amerinds, Central American"[Title/Abstract]) OR ("Indians, South American"[Title/Abstract]) OR (Tribes[Title/Abstract]) OR (indigen[Title/Abstract]) OR (aborigine[Title/Abstract]) OR (tribal[Title/Abstract]) OR ("native people"[Title/Abstract])).

Final strategy: Maternal and congenital syphilis in Indigenous Peoples (((#1 AND #2) OR #3) AND #4).

Biblioteca Digital de Teses e Dissertação (BDTD)
Maternal syphilis: (#1 AND #2).
Strategy#1: (syphilis OR "treponemal infections" OR treponema OR LUES)

Strategy#2: (pregnancy OR "Pregnancy Complications, Infectious" OR pregnant* OR maternal* OR maternity OR mothers OR mother* OR women OR woman OR parturient* OR gestational OR prenatal* OR antenatal OR "vertical transmission" OR "Vertical Infection" OR "congenital" OR "mother-to-child transmission" OR "Infectious Disease Transmission, Vertical")

Congenital syphilis (#3)
Strategy#3: ("Syphilis, Congenital" OR "congenital syphilis")

Indigenous Peoples (#4)
Strategy#4: ("Indigenous Peoples" OR "Population Groups" OR "Indigenous culture" OR "indigenous population" OR "Health services, indigenous" OR "Oceanic Ancestry Group" OR "Alaska natives" OR "indigenous population" OR "Continental Population Groups" OR "Health of Indigenous People" OR "Native Americans" OR "Inuits" OR "First Nations" OR "Amerinds" OR tribes OR indigen* OR aborigine OR tribal OR "native people")

Final strategy: Maternal and congenital syphilis in Indigenous Peoples (((#1 AND #2) OR #3) AND #4).

Biblioteca Virtual em Saúde (BVS/MS) e Biblioteca Virtual em Saúde – Saúde Indígena (BVS-Saúde Indígena)
Maternal syphilis: (#1 AND #2).
Strategy#1: (syphilis OR "treponemal infections" OR treponema OR LUES)

Strategy#2: (pregnan* OR maternal* OR maternity OR mother* OR women OR woman OR parturient* OR gestational OR prenatal* OR antenatal OR "Vertical Infection" OR congenital)

Congenital syphilis (#3)
Strategy#3: ("Syphilis, Congenital" OR "congenital syphilis")

Indigenous Peoples (#4)
Strategy#4: ("indigenous people")

Final strategy: Maternal and congenital syphilis in Indigenous Peoples (((#1 AND #2) OR #3) AND #4).

Notes: The search was carried out in titles, abstracts, and keywords, using terms in English, Portuguese, and Spanish, whenever applicable.
