# Supplementary material for: Maternal and congenital syphilis in Indigenous Peoples: a scoping review of the worldwide literature
Source: Int J Equity Health. 2023 May 9;22:84. doi: 10.1186/s12939-023-01890-x (PMC10169209; doi:10.1186/s12939-023-01890-x)
Supplement: Supplementary file 2 — Additional File 2: Sources excluded following full-text review. [file 12939_2023_1890_MOESM2_ESM.pdf]

Additional File 2: Sources excluded following full-text review

N	Title	Author, Year	Publication (jornal, site)	Type	Reason for exclusion
1	Conocimientos, Actitudes y Prácticas sobre Prevención de Sífilis y VIH de Indígenas, Distrito Dr. Juan Eulogio Estigarribia, año 2014.	Alfonso, 2016	Rev. Inst. Med. Trop	SP	1
2	Detecção de sífilis adquirida em comunidades de difícil acesso da região Amazônia: desafio a ser superado com a utilização dos testes rápidos	Benzaken, 2009	ENSP, Fiocruz	PhD	1
3	Expansion of HIV and syphilis into the Peruvian Amazon: a survey of four communities of an indigenous Amazonian ethnic group	Bartlett, 2008	Int. J. Infect. Dis.	SP	1
4	Fulfilling prophecy? Sexually transmitted infections and HIV in Indigenous people in Western Australia.	Wright, 2005	Med. J. Aust.	SP	1
5	Hepatitis B virus, syphilis, and HIV seroprevalence in pregnant women and their male partners from six indigenous populations of the Peruvian Amazon Basin, 2007-2008	Ormaeche,2012	Int. J. Infect. Dis.	SP	1
6	Reproductive health, infertility and sexually transmitted infections in Indigenous women in a remote community in the Northern Territory.	Kildea, 2000	Aust N Z Public Health	SP	1
7	Sexually Transmitted Diseases Among American Indians in Arizona: An Important Public Health Disparity.	Winscott, 2010	Public Health Rep	SP	1
8	Was infectious syphilis being misclassified in remote Australian outbreaks? Evidence that informed modification of the national case definition	Preston-Thomas, 2015	Commun Dis Intell	SP	1
9	Condom use among Aboriginal people in Ontario, Canada.	Calzavara, 1998	Int J STD AIDS	SP	1
10	Human papillomavirus and other genital infections in indigenous women from Paraguay: a cross-sectional analytical study.	Mendoza, 2013	BMC Infect Dis	SP	1
11	Incident sexually transmitted infections and their risk factors in an aboriginal community in Australia: A population based cohort study.	Miller, 2001	Sex Transm Infect	SP	1
12	Indigenous healers' beliefs and practices concerning sexually transmitted diseases.	Mulaudzi, 2006	Curationis	SP	1
13	Diagnosis of Treponema pallidum and HIV infections and contact tracing in the Mbyá-Guaraní population of the Puerto Iguazú, Argentina. A cross-sectional study.	Marx, 2019	Trans R Soc Trop Med Hyg	CA	4
14	Congenital syphilis - United States, 2003-2008.	CDC, 2010	MMWR, CDC	RP	3
15	Epidemiology of an outbreak of infectious syphilis in Alberta.	Romanowski, 1991	Int J STD AIDS	SP	1
16	Examination of a prenatal syphilis screening program, Alberta, Canada: 2010-2011	Plitt, 2016	Can J Public Health	SP	3
17	Hepatitis B virus, human immunodeficiency virus type-1, and syphilis among women attending prenatal clinics in Belize, Central America.	Scott-Wright, 1997	AM J Trop Med Hyg	SP	1
18	HIV and syphilis serostatus of antenatals in traditional Maasai pasturalist communities in Kajiado District, Kenya: 1989-1992.	Valadez, 1999	Trop Doct	SP	1
19	Home-based counseling and testing for HIV and syphilis - an evaluation of acceptability and quality control, in remote Amazonas State, Brazil.	Ribeiro, 2015	Sex Transm Infect	SP	1
20	Sexually transmitted diseases in Australia: a decade of change. Epidemiology and surveillance.	Mulhall, 1995	Ann Acad Med Singap	SP	1
21	Syphilis epidemiology and public health interventions in Western Australia from 1991 to 2009.	Kwan, 2012	Sex Health	SP	1
22	The Co-occurrence of Intimate Partner Violence and Syphilis among Pregnant Women in Bolivia.	Díaz-Olavarrieta, 2009	J Womens Health	SP	1
23	Surveillance for gonorrhea and primary and secondary syphilis among adolescents, United States--1981-1991.	Webster, 1993	MMWR, CDC	RP	1
24	High prevalence of HIV and syphilis in a remote native community of the Peruvian Amazon.	Zavaleta, 2007	Am J Trop Med Hyg.	SP	1
25	High Prevalence of Sexually Transmitted Infections, and High-Risk Sexual Behaviors Among Indigenous Adolescents of the Comarca Ngäbe-Buglé, Panama.	Gabster, 2019	Sexually Transmitted Diseases	SP	1
26	HIV, Chlamydia, Gonorrhea, and Primary and Secondary Syphilis among American Indians and Alaska Natives Within Indian Health Service Areas in the United States, 2007-2010.	Walker, 2015	J Community Health	SP	1
27	HIV, syphilis, and viral hepatitis among Latin American indigenous peoples and Afro-descendants: a systematic review.	Russel, 2019	Rev Panam Salud Publica	REV	2
28	Improving the accuracy of Aboriginal and non-Aboriginal disease notification rates using data linkage.	Mak, 2008	BMC Health Services Research	SP	1

29	Increasing trends of sexually transmitted infections among American Indian/ Alaska natives, 2013-2017	Apostolou, 2020	Sexually Transmitted Diseases	CA	1
30	La treponematosis precolombina en Cuba.	Canosa, 2004	Revista Médica Electrónica	SP	1
31	Pinta, yaws, and venereal syphilis in Colombia.	Hopkins, 1977	Int J Epidemiol.	SP	1
32	Prevalence and Correlates of a Diagnosis of Sexually Transmitted Infection Among Young Aboriginal and Torres Strait Islander People: A National Survey.	Ward, 2016	Sexually Transmitted Diseases	SP	1
33	Prevalence of chlamydia, gonorrhea, syphilis, and trichomonas in Aboriginal and Torres Strait Islander Australians: a systematic review and meta-analysis.	Graham, 2016	Sexual Health	REV	2
34	Prevalence of HIV and syphilis and social behavior characteristics of native populations in Three Geographical Areas of Paraguay – 2011.	Aguilar, 2012	Int. J. Infect. Dis.	CA	1
35	Prevalence of Syphilis and Related Risk Behaviors Among Women in 5 Distinct Indigenous Populations in Paraguay	Aguilar, 2019	Sexually Transmitted Diseases	SP	1
36	Prevalência de anticorpos contra os agentes causadores da hepatite, malária, sífilis e toxoplasmose em cinco populações humanas distintas da Amazonia Brasileira.	Ferrazoni, 1982	Rev. lnst. Med. trop. São paulo	SP	1
37	Prevalencia de sífilis y características del comportamiento de los jóvenes indígenas del Paraguay, 2016	Ortiz, 2018	Mem. Inst. Investig. Cienc. Salud	SP	1
38	Racial misclassification of American Indians in Oklahoma State surveillance data for sexually transmitted diseases.	Thoroughman, 2002	Am J Epidemiol	SP	1
39	Doenças sexualmente transmissíveis e aids entre indígenas do Distrito Sanitário Especial Indígena do Mato Grosso do Sul de 2001 a 2005	Ferri, 2011	Saúde Coletiva	SP	1
40	Prevalência de infecção por HIV, HTLV, VHB e de sífilis e clamídia em gestantes numa unidade de saúde terciária na Amazônia ocidental brasileira	Machado Filho, 2010	Rev. Bras. Ginecol. Obstet.	SP	1
41	Syphilis in pregnancies and vertical transmission as a public health problem	Loureiro, 2012	J Nurs	SP	1
42	`Brothel of the Pacific': Syphilis and the Urban Regulation of Laikini Wahine in Honolulu, 1855-75	Kindell, 2019	Journal Pacific History	SP	1
43	Pre-pregnancy predictors linked to miscarriage among Aboriginal and Torres Strait Islander women in North Queensland	Campbell, 2011	Australian and New Zealand Journal of Public Heatlh	SP	1
44	Field performance of a rapid point-of-care diagnostic test for antenatal syphilis screening in the Amazon region, Brazil	Benzaken, 2011	International Journal of STD & AIDS	SP	3
45	Diagnosis of Treponema pallidum and HIV infections and contact tracing in the Mbyá-Guaraní population of the Puerto Iguazú, Argentina. A cross sectional study.	Marx, 2019	Trans R Soc Trop Med Hyg	CA	4

Publication type: **MD=**Master dissertation**;** **PhD=**PhD thesis; **SP**=Scientific paper; **CA**=Conference/Congress abstract**;** **RP**=Report**;** **REV=** Review (Systematic, others, meta-analysis)**;** **Exclusion criteria:** **1=**Does not analyze congenital or gestational syphilis; **2=** Results of systematic review without additional or other contributions; **3=**No specific results for indigenous people; **4**= Publication with same data of another selected study.
