# Supplementary material for: Maternal and congenital syphilis in Indigenous Peoples: a scoping review of the worldwide literature
Source: Int J Equity Health. 2023 May 9;22:84. doi: 10.1186/s12939-023-01890-x (PMC10169209; doi:10.1186/s12939-023-01890-x)
Supplement: Supplementary file 3 — Additional File 3: Data extraction instrument. [file 12939_2023_1890_MOESM3_ESM.pdf]

Additional File 3: Data extraction instrument

<table>
<tr><td colspan="3">DATA EXTRACTION FORM – SCOPING REVIEW ON MATERNAL AND CONGENITAL SYPHILIS IN INDIGENOUS PEOPLES, 2021</td></tr>
<tr><td colspan="3">ARTICLE IDENTIFICATION:</td></tr>
<tr><td colspan="3">Title of the article:</td></tr>
<tr><td colspan="3">Aim/purpose of the study:</td></tr>
<tr><td colspan="3">Publication Journal:</td></tr>
<tr><td colspan="2">First Author:</td><td>Number of authors:</td></tr>
<tr><td colspan="3">Institutional affiliation of the first author:
Institutional affiliation of the last author:</td></tr>
<tr><td colspan="3">Publication year:</td></tr>
<tr><td colspan="3">Data collection year:</td></tr>
<tr><td colspan="3">Publication type; journal; publication language:</td></tr>
<tr><td colspan="3">Study design: DCS=Descriptive, case series; CS=Cross-Sectional/Seroprevalence; E=Ecological, descriptive using surveillance data; D=Descriptive, outbreaks; CC=Case-Control; CH=Cohort; SR=Systematic review or meta-analysis; CT=Community trial; VR=Validity/reliability; CE=Costs-effectiveness (screening); QLQ=Qualitative/Quantitative (Case study and/or cross-sectional);</td></tr>
<tr><td colspan="3">Study population (include all existing details - age range, area of residence):</td></tr>
<tr><td colspan="3">Size of the study population (n):</td></tr>
<tr><td colspan="3">Continent; region (1-South America; 2-Central America; 3-North America; 4-Africa; 5-Asia; 6-Oceania):</td></tr>
<tr><td colspan="3">Country:</td></tr>
<tr><td colspan="3">Detailed location (region, state, county, etc.):</td></tr>
<tr><td>If in Brazil: Region</td><td>UF:</td><td>County/City:</td></tr>
<tr><td>If in Brazil: Indigenous Sanitary District</td><td>Indigenous Land:</td><td>Other informations:</td></tr>
<tr><td colspan="3">Race/color and ethnicity:</td></tr>
<tr><td colspan="3">Source of information on race/color and ethnicity (secondary database – Health Information Systems (HIS), medical records, registries, etc.; primary data - interviews, others):</td></tr>
<tr><td colspan="3">Way of assigning race and/or ethnicity to the pregnant woman or child (self-declaration, heteroclassification):</td></tr>
<tr><td colspan="3">Topics covered: HT=Historical report of gestational and congenital syphilis in indigenous populations; DQ=Data quality related to congenital and gestational syphilis in indigenous peoples; DAH= Diagnostic, provision, access, and use of health services related to gestational and congenital syphilis in indigenous peoples; DF=Incidence and prevalence of congenital and gestational syphilis in indigenous populations and health inequities; DOC=Determinants of outcomes of congenital syphilis in indigenous populations; DT=Determinants of congenital or gestational syphilis in indigenous populations; and IP=Impacts of social and health public policies on congenital and gestational syphilis in indigenous populations.</td></tr>
<tr><td colspan="3">Statistical analysis model and relevant aspects of the method:</td></tr>
<tr><td colspan="3">Ethical aspects:</td></tr>
<tr><td colspan="3">Main results:</td></tr>
<tr><td colspan="3">Determinants/risk factors/risk markers/Intervention studied and outcome of associations (frequency measures, crude, adjusted associations, point estimates and CIs, and adjustment variables):</td></tr>
<tr><td colspan="3">Critical analysis:</td></tr>
<tr><td colspan="3">Comments/Observations:</td></tr>
<tr><td colspan="3">Date of extraction:</td></tr>
<tr><td colspan="3">Responsible for extraction:</td></tr>
</table>
